# Supplementary material for: c-Abl Phosphorylates MFN2 to Regulate Mitochondrial Morphology in Cells under Endoplasmic Reticulum and Oxidative Stress, Impacting Cell Survival and Neurodegeneration
Source: Antioxidants (Basel). 2023 Nov 16;12(11):2007. doi: 10.3390/antiox12112007 (PMC10669615; doi:10.3390/antiox12112007)
Supplement: Supplementary file 1 [file antioxidants-12-02007-s001.zip › antioxidants-2639459-supplementary.pdf]

Figure S1

A

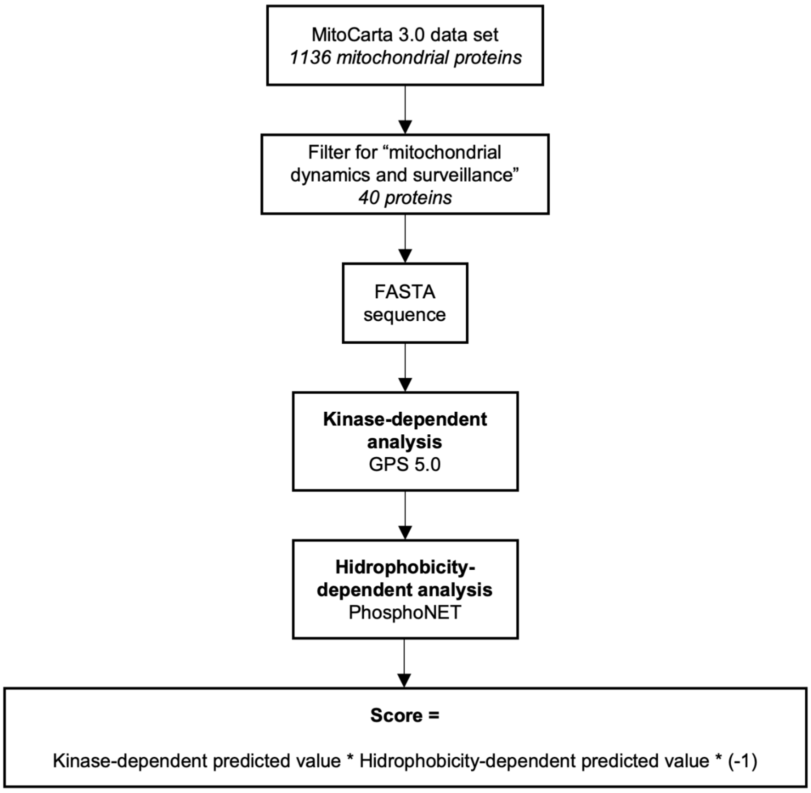

B

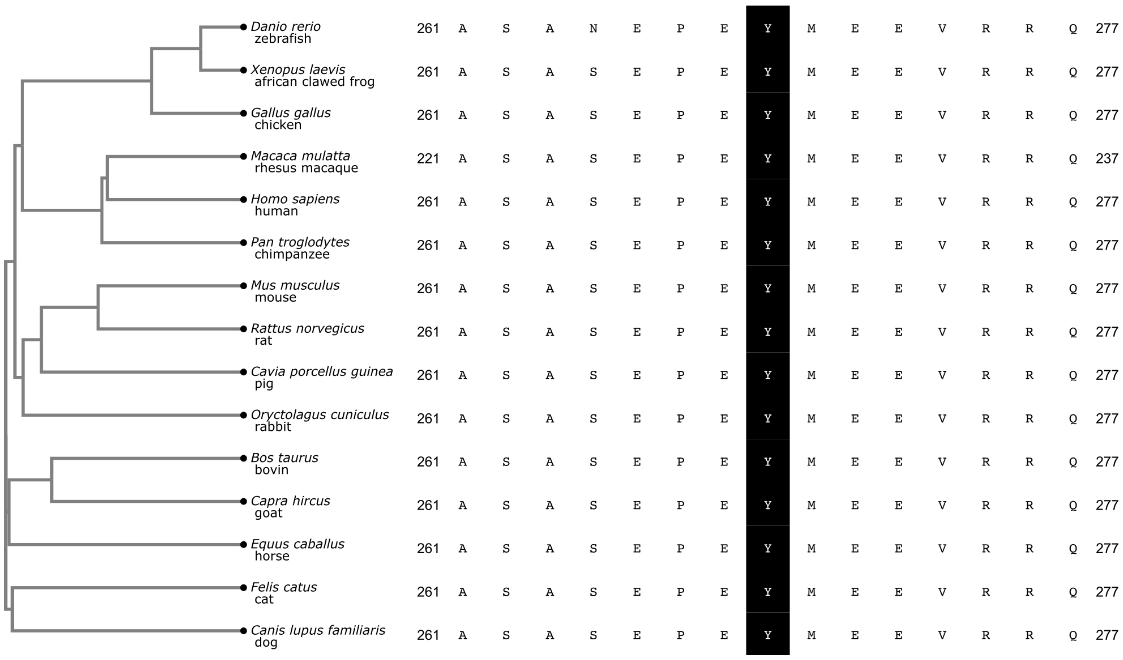

		-7	-6	-5	-4	-3	-2	-1	0	+1	+2	+3	+4	+5	+6	+7	
Danio rerio zebrafish	261	A	S	A	N	E	P	E	Y	M	E	E	V	R	R	Q	277
Xenopus laevis african clawed frog	261	A	S	A	S	E	P	E	Y	M	E	E	V	R	R	Q	277
Gallus gallus chicken	261	A	S	A	S	E	P	E	Y	M	E	E	V	R	R	Q	277
Macaca mulatta rhesus macaque	221	A	S	A	S	E	P	E	Y	M	E	E	V	R	R	Q	237
Homo sapiens human	261	A	S	A	S	E	P	E	Y	M	E	E	V	R	R	Q	277
Pan troglodytes chimpanzee	261	A	S	A	S	E	P	E	Y	M	E	E	V	R	R	Q	277
Mus musculus mouse	261	A	S	A	S	E	P	E	Y	M	E	E	V	R	R	Q	277
Rattus norvegicus rat	261	A	S	A	S	E	P	E	Y	M	E	E	V	R	R	Q	277
Cavia porcellus guinea pig	261	A	S	A	S	E	P	E	Y	M	E	E	V	R	R	Q	277
Oryctolagus cuniculus rabbit	261	A	S	A	S	E	P	E	Y	M	E	E	V	R	R	Q	277
Bos taurus bovin	261	A	S	A	S	E	P	E	Y	M	E	E	V	R	R	Q	277
Capra hircus goat	261	A	S	A	S	E	P	E	Y	M	E	E	V	R	R	Q	277
Equus caballus horse	261	A	S	A	S	E	P	E	Y	M	E	E	V	R	R	Q	277
Felis catus cat	261	A	S	A	S	E	P	E	Y	M	E	E	V	R	R	Q	277
Canis lupus familiaris dog	261	A	S	A	S	E	P	E	Y	M	E	E	V	R	R	Q	277

Figure S1. In silico analysis to determine potential c-Abl mitochondrial targets.

(A) Workflow of the in silico analysis in which 1136 proteins from the MitoCarta 3.0 data set where filtered under the "mitochondrial dynamic and surveillance" pathway, resulting in 40 proteins, from which FASTA sequence was obtained in UniProt data base and run for c-Abl kinase dependent analysis in the GPS 5.0 software with a medium cut-off. The residues that had a bigger value that cut off value were searched in the PhosphoNET online tool for Hydrophobicity- dependent analysis. The score was calculated as the multiplication between the kinase-dependent predicted value, the hydrophobicity-dependent value and -1 to correct the negative values of the hydrophobicity analysis.

(B) Y269 in MFN2 is conserved in a wide range of species from which a phylogenetic tree is shown.

Figure S2

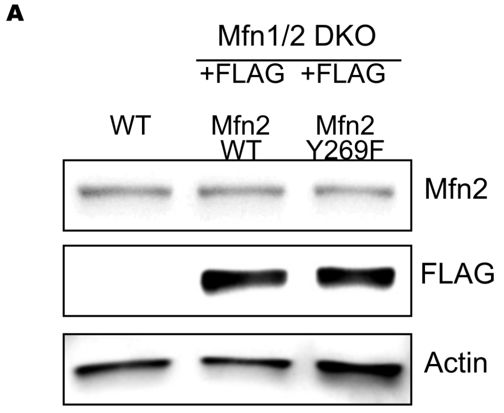
A
Mfn1/2 DKO
+FLAG
+FLAG
WT
Mfn2 WT
Mfn2 Y269F
Mfn2
FLAG
Actin

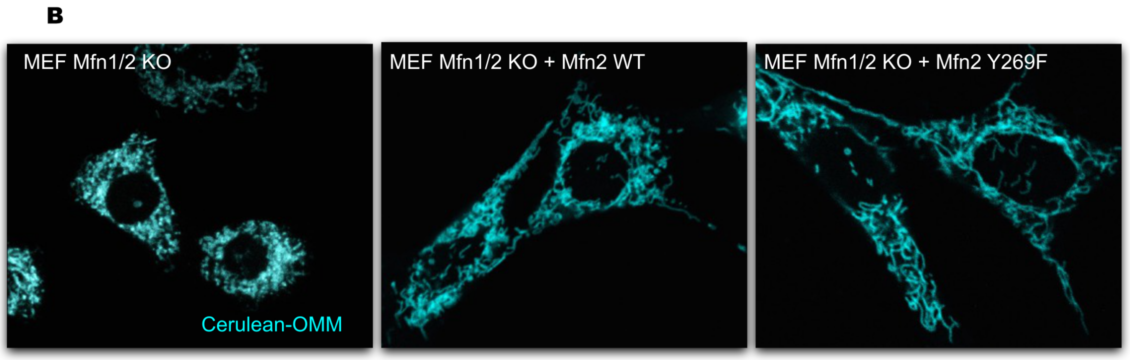
B
MEF Mfn1/2 KO
Cerulean-OMM
MEF Mfn1/2 KO + Mfn2 WT
MEF Mfn1/2 KO + Mfn2 Y269F

Figure S2. Stable overexpression of FLAG-Mfn2 restitutes the mitochondrial morphology in Mfn1/2- deficient MEF cells.
(A) Protein levels of MFN2 and FLAG in stable expression of MFN2 variants in Mfn1/2-deficient MEF cells.
(B) Microscopy for cerulean-OMM marker (cyan) showing mitochondrial morphology in stable expression of MFN2 variants in Mfn1/2- deficient MEF cells.

Figure S3

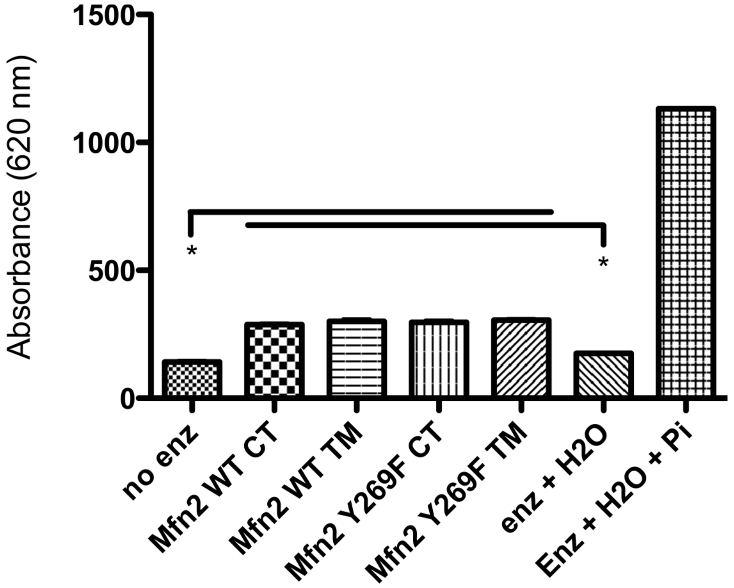

Absorbance (620 nm)
1500
1000
500
0
*
*
no enz
Mfn2 WT CT
Mfn2 WT TM
Mfn2 Y269F CT
Mfn2 Y269F TM
enz + H2O
Enz + H2O + Pi

Figure S3. GTP hidrolytic rate is not altered in mutant Mfn2.

GTP assay detecting released Pi at 620 nm from immunoprecipitated Mfn2-FLAG

Figure S4

A

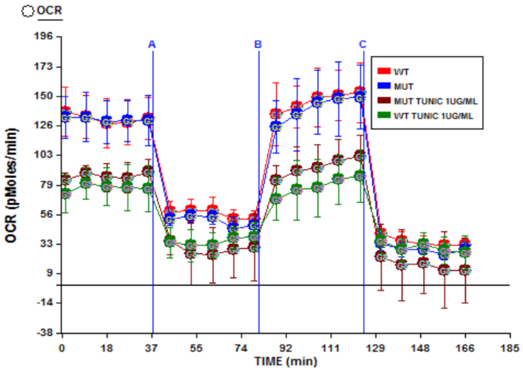

B

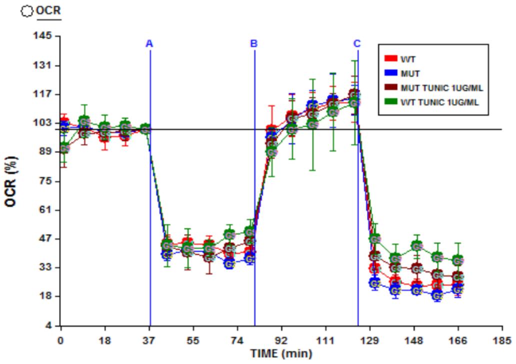

C

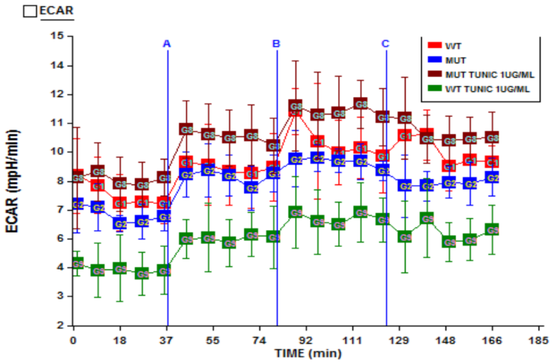

A: Oligomycin (0.5uM)
B: FCCP (0.5uM)
C: Rotenone (0.5uM) + Antimycin A (1uM)

Figure S5. Mitochondrial activity in MFN WT and Y269F mutant MEF

(A) OCR in MFN WT and Y269F mutant MEF expressed as pMoles/mln.

(B) OCR in MFN WT and Y269F mutant MEF expressed as percentage (%) respect to basal condition.

(C) ECAR in MFN WT and Y269F mutant MEF expressed as mpH/mln.

Figure S5

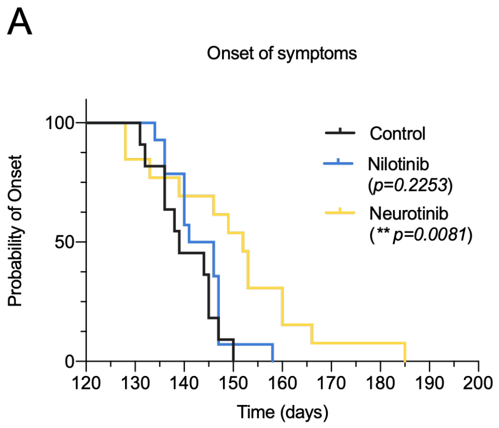

A
Onset of symptoms
Probability of Onset
100
50
0
120 130 140 150 160 170 180 190 200
Time (days)
Control
Nilotinib
(p=0.2253)
Neurotinib
(** p=0.0081)
B
Death
Probability of Survival
100
50
0
120 130 140 150 160 170 180 190 200
Time (days)
Control
Nilotinib
(p=0.8548)
Neurotinib
(p=0.1237)

Figure S5. Pharmacological inhibition of c-Abl improves ALS phenotype in SOD1 G93A mice.

(A) Kaplan-Meier curve shows the probability of onset of symptoms in control-fed (black line,n=11), nilotinib-fed (blue line, n=14) and neurotinib-fed (yellow line, n=13) SOD1 G93A mice using log-rank (Mantel-Cox) test.
(B) Kaplan-Meier survival curve shows the probability of death in the same groups of mice using log-rank (Mantel-Cox) test.
